# Estrogen Prevents Oxidative Damage to the Mitochondria in Friedreich's Ataxia Skin Fibroblasts

**DOI:** 10.1371/journal.pone.0034600

**Published:** 2012-04-03

**Authors:** Timothy E. Richardson, Amanda E. Yu, Yi Wen, Shao-Hua Yang, James W. Simpkins

**Affiliations:** Institute for Aging and Alzheimer's Disease Research, Department of Pharmacology and Neuroscience, University of North Texas Health Science Center, Fort Worth, Texas, United States of America; University of Windsor, Canada

## Abstract

Estrogen and estrogen-related compounds have been shown to have very potent cytoprotective properties in a wide range of disease models, including an *in vitro* model of Friedreich's ataxia (FRDA). This study describes a potential estrogen receptor (ER)-independent mechanism by which estrogens act to protect human FRDA skin fibroblasts from a BSO-induced oxidative insult resulting from inhibition of *de novo* glutathione (GSH) synthesis. We demonstrate that phenolic estrogens, independent of any known ER, are able to prevent lipid peroxidation and mitochondrial membrane potential (ΔΨm) collapse, maintain ATP at near control levels, increase oxidative phosphorylation and maintain activity of aconitase. Estrogens did not, however, prevent BSO from depleting GSH or induce an increased expression level of GSH. The cytoprotective effects of estrogen appear to be due to a direct overall reduction in oxidative damage to the mitochondria, enabling the FRDA fibroblast mitochondria to generate sufficient ATP for energy requirements and better survive oxidative stress. These data support the hypothesis that phenol ring containing estrogens are possible candidate drugs for the delay and/or prevention of FRDA symptoms.

## Introduction

First reported in 1863 by Nikolaus Friedreich [1], [2], [3], Friedreich's Ataxia (FRDA) has an incidence of 1∶50,000–1∶20,000 and a carrier rate of 1∶120–1∶60 in the Caucasian population of the United States, making it the most prevalent form of hereditary ataxia [4], [5], [6], [7]. This disorder is inherited in an autosomal recessive manner caused by a GAA repeat expansion in the first intron of the FXN gene on chromosome 9q13-21 [8], [9], causing a self-associating complex of sticky DNA to form, hindering transcription [10] and significantly reducing the expression of Frataxin [11], [12], [13], [14]. The number of GAA repeats on the smaller allele is inversely proportional to the intracellular levels of Frataxin [15] and positively correlated to the severity of patient symptoms [16], [17]. Although the exact role of Frataxin is currently unclear, its loss has two direct effects in several reported tissue types: impaired formation of iron-sulfur (Fe-S) clusters and a rise in intracellular reactive oxygen species (ROS) [6], [7], [14], [18]. The decrease in Fe-S containing proteins, such as heme, electron transport chain (ETC) complexes I–III and the Kreb's cycle protein aconitase severely impairs cellular respiration [14], [19], [20], [21], which is further complicated by simultaneous oxidative damage to these mitochondrial proteins [13], [18], [21], [22], [23]. These events all culminate in an inability of the mitochondria to fulfill the cell's energy requirements resulting in cell death [14], a mechanism of death common to many neurodegenerative diseases (for review see Refs. [24], [25]).

First established more than a decade ago [26], [27], the neuro- and cytoprotective effects of 17β-Estradiol (E2) are well known. However the exact mechanisms remain elusive [24]. There are now numerous reports showing that estrogen and estrogen-like compounds are effective in protecting against a wide variety of insults in numerous different cell types [28], including human Friedreich's ataxia skin fibroblasts [29]. Much current research focuses on the mitochondrial mechanisms of estrogen neuroprotection [30]. It is known that ERα and ERβ localize to the mitochondria in many different cell types, including cerebrovascular cells, primary neurons, cardiomyocytes and hippocampal cell lines [31], [32]. E2 upregulates expression of genes necessary for oxidative phosphorylation encoded in both nuclear and mitochondrial DNA, elevating levels of these complexes and enhancing aerobic ATP production [31]. E2 has been shown to provide neuroprotection through the modulation of calcium flux in the cell and calcium sequestration by the mitochondria in primary hippocampal cells [33], [34] and by increasing the expression of the anti-apoptotic protein, Bcl-2 [33]. Estrogens have also been shown to act on pro-survival pathways including ERK, CREB and MAPK [35], and to have direct and indirect antioxidant effects [24], [29], [30].

In a previous study, we showed that several estrogen-like compounds are extremely potent and efficacious cytoprotectants of human FRDA fibroblasts against L-buthionine (S,R)-sulfoximine (BSO)-induced oxidative stress independent of any known estrogen receptor (ER) [29]. This effect appears to be dependent on the presence of at least one phenol ring in the molecular structure of the compound and is at least in part due to antioxidant properties and the attenuation of reactive oxygen species [29], a strategy previously investigated with other potential antioxidants [6], [14], [36], [37], [38]. However, as in other cell and animal disease models, the precise mechanism of estrogen action in Friedreich's ataxia is not yet fully understood.

In this study, we investigate the mechanism of estrogen action in human FRDA skin fibroblasts. Using BSO to induce oxidative stress, we show that all phenolic estrogen-like compounds tested are able to attenuate ROS production [29], prevent lipid peroxidation and maintain mitochondrial function. This occurs without the prevention of BSO-induced reduction of glutathione (GSH). These effects are also independent of any known ER. These data presented here indicate that estrogens effectively prevent pro-oxidant stress in the mitochondria [6], [36], [37] by preventing the excess ROS associated with FRDA from damaging mitochondrial enzymes and inducing cell death.

## Methods

### Cell Culture

Fibroblasts from a 30 year old FRDA patient, obtained from Coriell Institute (Camden, NJ, USA) were maintained in Dulbecco's Modified Eagle Medium (DMEM; ThermoScientific, Waltham, MA, USA) with 10% charcoal-stripped fetal bovine serum (FBS; ThermoScientific), 1% GlutaMAX (ThermoScientific) and 1% penicillin-streptomycin (Invitrogen, Carlsbad, CA, USA) at 37°C, 5% CO_2_ and 90% humidity. Before vehicle or BSO treatment, FRDA fibroblast media was changed to phenol red- and sodium pyruvate-free DMEM (ThermoScientific) containing 1% penicillin-streptomycin. All experiments were conducted with FRDA cells from passage 14–21.

### Chemicals & Reagents

**Figure 1:**
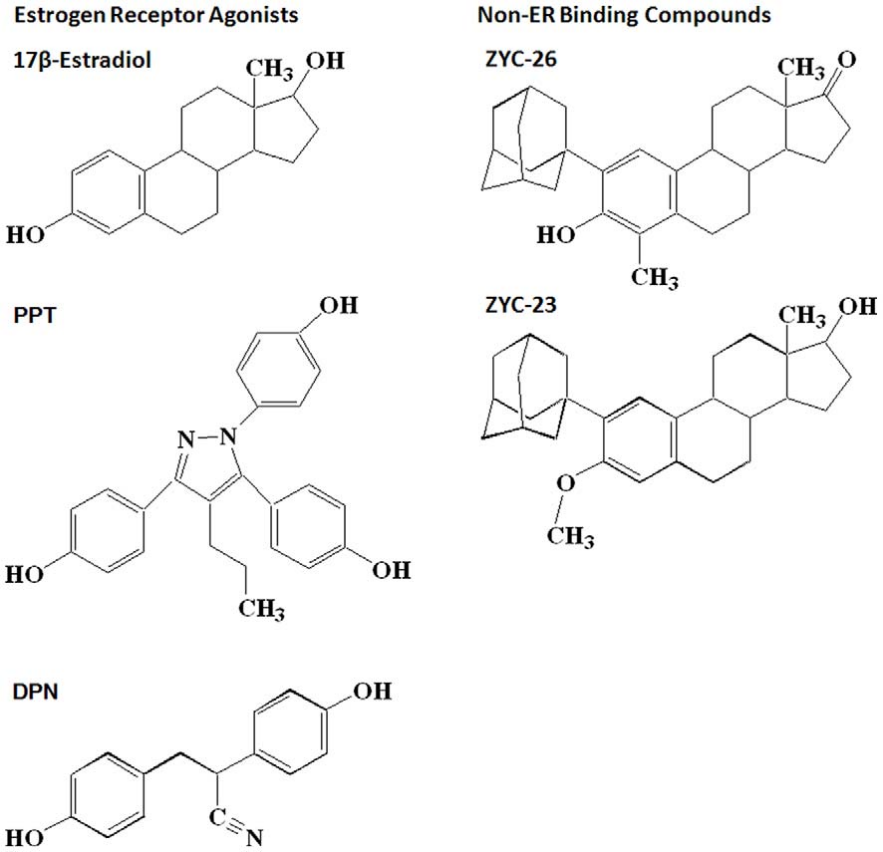
Structures of compounds assessed for protection against BSO toxicity in FRDA fibroblasts.

17β-Estradiol (E2) was obtained from Steraloids, Inc. (Newport, RI, USA). L-buthionine (S,R)-sulfoximine (BSO) was acquired from Sigma-Aldrich (St Louis, MO, USA). ICI 182,780, 4,4′,4′-(4-Propyl-[1H]-pyrazole-1,3,5-triyl)trisphenol (PPT) and diarylpropionitrile (DPN) were purchased from Tocris Bioscience (Ellisville, MO, USA). ZYC-26 and ZYC-23 were synthesized in the Covey laboratory [39]. Structures for these steroids were drawn using ChemDraw software (CambridgeSoft, Cambridge, MA), and are provided in Fig. 1.

### Steroid Treatment

FRDA fibroblasts were plated in 24- or 96-well plates at a density of 3,000–35,000 cells per well in DMEM with 10% FBS, 1% GlutaMAX and 1% penicillin-streptomycin. After 24 hours the growth media was removed and replaced with the phenol red- and sodium pyruvate-free DMEM. The cells were then treated for 12–48 hours, depending on the assay, with either dimethyl sulfoxide vehicle control (DMSO; Sigma-Aldrich, St Louis, MO, USA) or 1 mM BSO in the presence of 100 nM E2, DPN, PPT, ZYC-26 or ZYC-23, concentrations of estrogen-like molecules which have been shown to be neuroprotective in various cell lines [40] and cytoprotective in this FRDA fibroblast line [29].

### Calcein AM Cell Imaging

Cells were plated on a 96-well plate at a density of 5,000 cells per well, then treated with vehicle or 1 mM BSO. After 24, 36 and 48 hours of BSO treatment, the media was removed, and 1 µg/mL Calcein AM (CalBiochem, San Diego, CA, USA) in phosphate buffer pH 7.2 (PBS; Fisher Scientific, Pittsburg, PA, USA) was added to each well and the plate was incubated for 10 minutes at 37°C. The cells were then photographed using a Zeiss Axio Observer Z1 inverted microscope (Carl Zeiss MicroImaging, Thornwood, NY).

### Lactate Dehydrogenase (LDH) Cell Viability Assay

After 48 hours of treatment 50 µL of media was removed from each well of the 96-well plate and placed in a separate 96-well plate. 100 µL of a solution consisting of 12 mL of 200 mM pH 8.2 Tris(hydroxymethyl)aminomethane hydrochloride (Sigma-Aldrich, St Louis, MO, USA) with 50 µL lactic acid and 4.2 mg iodonitrotetrazolium chloride (INT; Sigma-Aldrich, St Louis, MO, USA), 1.1 mg phenazine methosulphate (PMS; Sigma-Aldrich, St Louis, MO, USA) and 10.8 mg β-nicotinamide adenine dinucleotide hydrate (NAD; Sigma-Aldrich, St Louis, MO, USA) was added to each well. The absorbance of the resulting reaction was read with a Tecan Infinite M200 plate reader at 490 nm and recorded once the reaction is linear for greater than 2 minutes. Cell viability for each well (WellX) was determined by: 100−(100 * (WellX - media)/(0.1%TritonX100-media)). Measurements were then confirmed by visual inspection of the FRDA fibroblasts.

### Western Blots

FRDA fibroblasts and 661W [41], [42] photoreceptor cells were grown in 10 cm plates until ∼80% confluent. The cells were then removed from the plates using rubber cell scrapers and sonicated in RIPA lysis buffer. A Lowry assay was run to determine protein concentration for normalization and 20 µg of protein was loaded into each western blot well. ERα and ERβ were detected using ERα (H-184) rabbit polyclonal IgG antibody and ERβ (H-150) rabbit polyclonal IgG antibody, obtained from Santa Cruz Biotechnology (Santa Cruz Biotechnology, Inc., Santa Cruz, CA, USA). GAPDH (6C5) mouse monoclonal IgG antibody, also obtained from Santa Cruz Biotechnology, was used as a control to ensure equivalent loading of protein loaded into each well.

### Lipid Peroxidation Assay

Cells were plated on 10 cm dishes and grown until ∼80% confluent. The cells were then treated with DMSO vehicle control or BSO and 100 nM E2, PPT, DPN, ZYC-26 or ZYC-23 in phenol red- and sodium pyruvate-free DMEM for 24 hours. At the end of treatment, the FRDA fibroblasts were removed from the plates using a rubber cell scraper and treated according to the Cayman 8-Isoprostane EIA protocol (Cayman Chemical Company, Ann Arbor, MI). The resulting absorbance was read on a Tecan Infinite F200 plate reader (Tecan Systems, Inc., San Jose, CA) at 340 nm.

### Aconitase Assay

Cells were plated in 10 cm dishes and grown until ∼80% confluent. The cells were then treated with DMSO vehicle control or BSO and 100 nM E2, PPT, DPN, ZYC-26 or ZYC-23 in phenol red- and sodium pyruvate-free DMEM for 24 hours. At the end of treatment, the FRDA fibroblasts were removed from the plates using a rubber cell scraper and treated according to the Cayman protocol. The resulting absorbance was read on a Tecan Infinite F200 plate reader at 340 nm.

### Mitochondrial Respiration Measurement

Human FRDA fibroblasts were plated in a 24-well Seahorse XF-24 assay plate at 35,000 cells/well and grown in FBS-containing DMEM media for 24 hours before being treated with either BSO or DMSO vehicle control and steroids for another 24 hours in FBS- and Phenol-red-free DMEM media. On the day of metabolic flux analysis, cells were changed to unbuffered DMEM media (DMEM base medium supplemented with 25 mM glucose, 10 mM sodium pyruvate, 31 mM NaCl, 2 mM GlutaMax, pH 7.4) and incubated at 37°C in a non-CO2 incubator for 1 hr. All media was adjusted to pH 7.4 on the day of assay. Eight baseline measurements of OCR and ECAR were taken before sequential injection of mitochondrial inhibitors, oligomycin (10 µM), FCCP (1 µM) and rotenone (5 µM). Four measurements were taken after each addition of mitochondrial inhibitor before injection of the next inhibitor. Oxygen consumption rate (OCR, an indicator of oxidative phosphorylation) and extracellular acidification rate (ECAR, an indicator of glycolysis) were automatically calculated and recorded by the Seahorse XF-24 software (Seahorse Bioscience, North Billerica, MA, USA).

### ATP Assay

Cells were plated on 10 cm dishes and grown until ∼80% confluent. The cells were then treated with DMSO vehicle control or BSO and 100 nM E2, PPT, DPN, ZYC-26 or ZYC-23 in phenol red- and sodium pyruvate-free DMEM for 24 hours. At the end of treatment, the FRDA fibroblasts were removed from the plates using a rubber cell scraper and treated according to the Abcam protocol (Abcam Inc., Cambridge, MA), then were read with an excitation of 535 nm and emission of 587 nm on a Tecan Infinite F200 plate reader.

### Mitochondiral Membrane Potential (ΔΨm) Measurement

Cells were plated on 96-well plates at a density of 3,000 cells/well. The cells were then treated with DMSO vehicle control or BSO and 100 nM E2, PPT, DPN, ZYC-26 or ZYC-23 in phenol red- and sodium pyruvate-free DMEM for 36 hours. A fluorescence resonance energy transfer (FRET) assay was used in this study to measure mitochondrial membrane potential collapse. In this assay, nonyl acridine orange (NAO; Enzo Life Sciences Inc., Plymouth Meeting, PA, USA) was used to stain cardiolipin in the inner mitochondrial membrane. Tetramethylrhodamine, ethyl ester, perchlorate (TMRE; AnaSpec Inc., Fremont, CA, USA) was added simultaneously with NAO to the cell culture to quench the NAO fluorescent signal, and incubated for 20 minutes at 37°C in the dark. As mitochondrial membrane potential collapsed at 36 hours after BSO treatment, TMRE was released from the mitochondria, allowing the NAO fluorescent signature to be read with an excitation of 495 nm and an emission of 519 nm with a Tecan Infinite F200 plate reader [43], [44], [45].

### Glutathione Assay

Cells were plated on 10 cm dishes and grown until ∼80% confluent. The cells were then treated with DMSO vehicle control or BSO and 100 nM E2, PPT, DPN, ZYC-26 or ZYC-23 in phenol red- and sodium pyruvate-free DMEM for 24 hours. At the end of treatment, the FRDA fibroblasts were removed from the plates using a rubber cell scraper and treated according to the Cayman protocol. The resulting absorbance was read on a Tecan Infinite F200 plate reader at 410 nm.

### Data and Statistics

All data are displayed as mean ±1 standard deviation. These data were analyzed using ANOVA against an alpha level of 0.05. All graphs were made using GraphPad Prism 5. For all groups, n = 8 wells and experiments were repeated three times to ensure consistency.

### Institutional Review Board for Human Samples

All samples that are accepted into the Coriell Institute NIGMS Human Genetic Cell Repository are collected with informed consent according to the Guidelines from OHRP at the time of collection, including the GM04078 line of human FRDA skin fibroblasts used in this manuscript. In addition to the review of all human subject requirements at the time of submission by a committee of individuals trained in human subject protection from research risk, the activities of the NIGMS Human Genetic Repository are reviewed annually by the Institutional Review Board of the Coriell Institute for Medical Research, represented by Lorraine H. Toji, Ph.D., Vice Chair Coriell Institutional Review Board. When samples are submitted, they are given a Repository number and no links to the individual subject are maintained.

## Results

### Human FRDA fibroblasts express small amounts of ERβ, but not ERα

**Figure 2:**
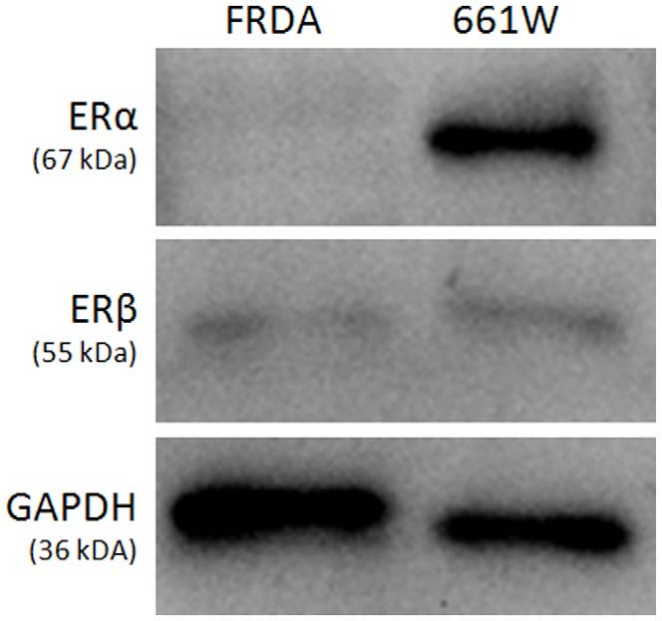
Western blot showing the presence of small amounts of ERβ and the absence of ERα in FRDA fibroblasts compared with 661W photoreceptor cells.

To determine the presence or absence of ERα and ERβ in human FRDA fibroblasts, western blots were run showing the intracellular presence of these proteins. Our western blots indicate the absence of ERα in human FRDA fibroblasts and the presence of very small amounts of ERβ compared with 661W cells (Fig. 2). These data agree with our previous pharmacological observations utilizing ICI 182,780 and ZYC compounds, showing that estrogen is likely not acting through any known ER to produce its effects on cell viability and instead acts to block production of free radicals [29].

### Timeline of events in BSO-induced FRDA fibroblast cell death

**Figure 3:**
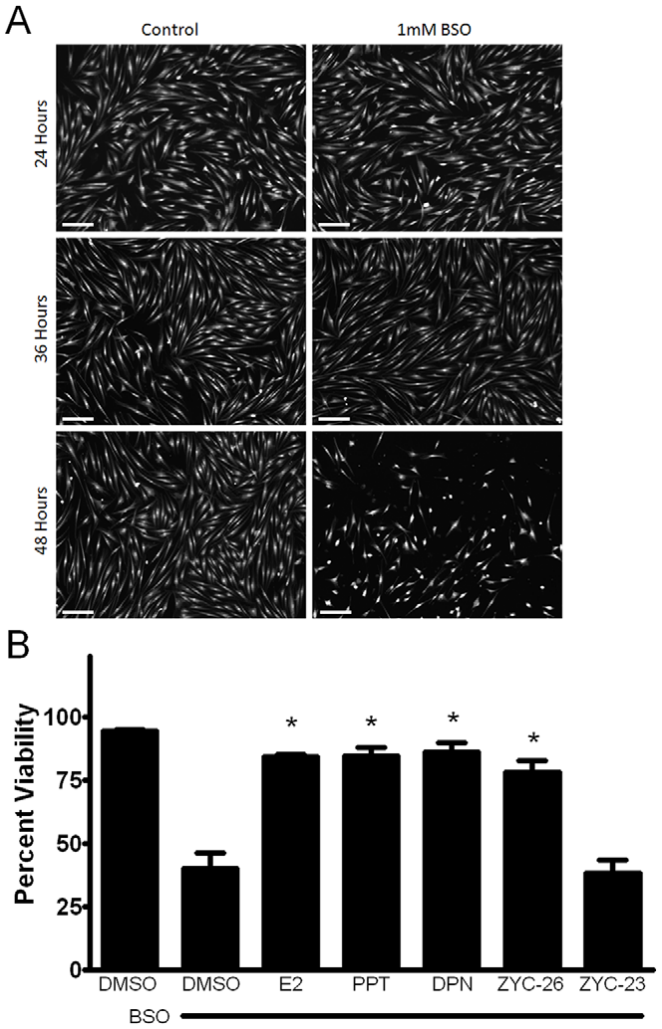
A.) Calcein AM imaging demonstrating cell viability between vehicle control and BSO treatment groups at 24, 36 and 48 hours. Scale bar = 200 µm. B.) Effects of E2, PPT, DPN, ZYC-26 and ZYC-23 on cell viability in BSO-treated FRDA fibroblasts. All steroid concentrations were 100 nM, DMSO concentration was 0.1% and BSO concentration was 1 mM. Depicted are mean ± SD for n = 8 per group. * indicated p<0.05 versus BSO alone-treated cells.

**Figure 4:**
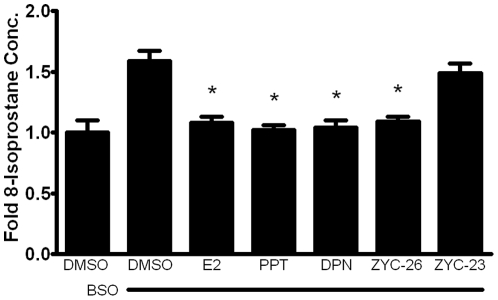
Effects of E2, PPT, DPN, ZYC-26 and ZYC-23 on intracellular lipid peroxidation in BSO-treated FRDA fibroblasts. All steroid concentrations were 100 nM, DMSO concentration was 0.1% and BSO concentration was 1 mM. Depicted are mean ± SD for n = 8 per group. * indicated p<0.05 versus BSO alone-treated cells. 1.0 normalized 8-isoprostane control concentration = 6.23 pg/mL.

**Figure 5:**
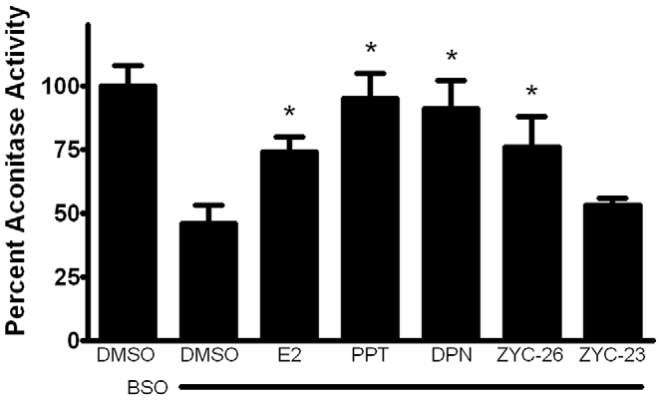
Effects of E2, PPT, DPN, ZYC-26 and ZYC-23 on the activity of aconitase in BSO-treated FRDA fibroblasts. All steroid concentrations were 100 nM, DMSO concentration was 0.1% and BSO concentration was 1 mM. Depicted are mean ± SD for n = 8 per group. * indicated p<0.05 versus BSO alone-treated cells.

**Figure 6:**
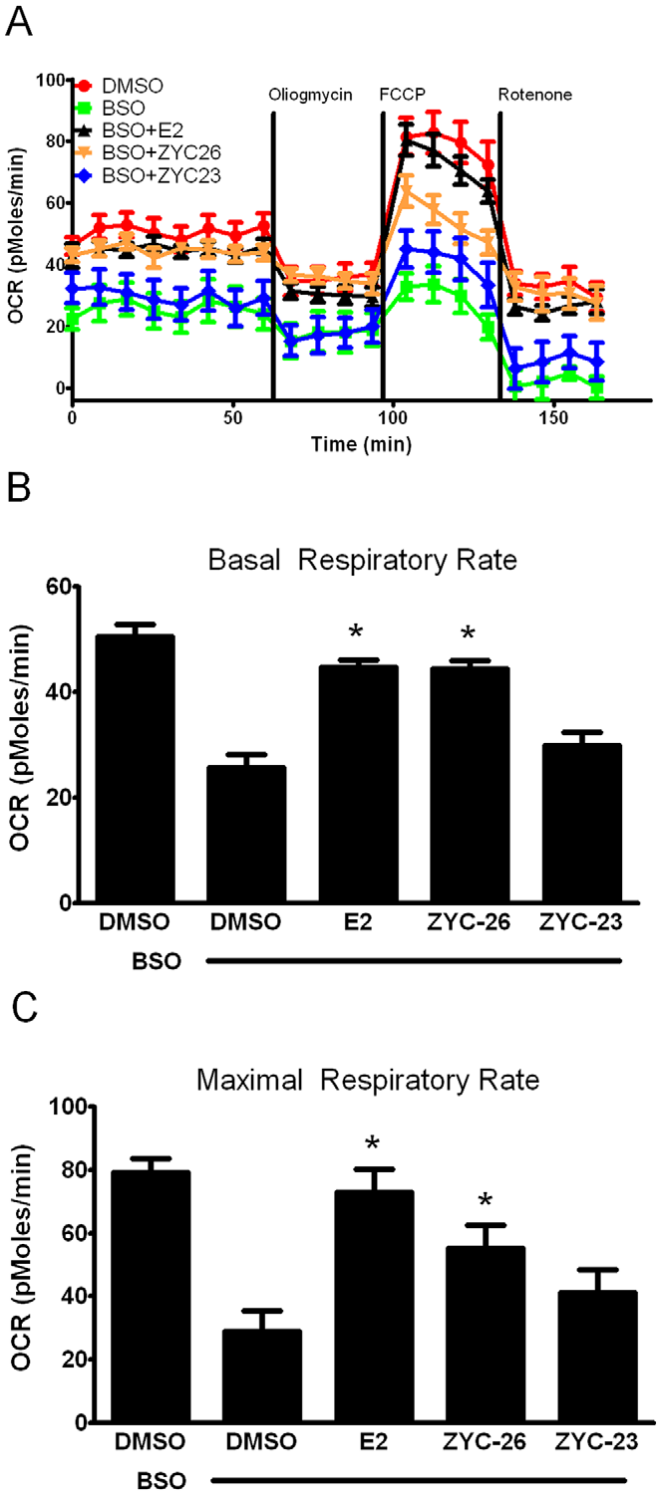
Effects of E2 and ZYC-26 on mitochondrial function in BSO-treated FRDA fibroblasts. A.) Oxygen consumption rate (OCR; in pMoles/min) B.) Basal respiratory rate (in pMoles/min) C.) Maximal respiratory rate (in pMoles/min) All steroid concentrations were 100 nM, DMSO concentration was 0.1% and BSO concentration was 1 mM. Depicted are mean ± SD for n = 8 per group. * indicated p<0.05 versus BSO alone-treated cells.

**Figure 7:**
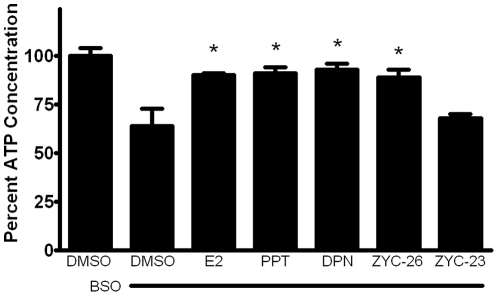
Effects of E2, PPT, DPN, ZYC-26 and ZYC-23 on the intracellular ATP concentration inside of BSO-treated FRDA fibroblasts. All steroid concentrations were 100 nM, DMSO concentration was 0.1% and BSO concentration was 1 mM. Depicted are mean ± SD for n = 8 per group. * indicated p<0.05 versus BSO alone-treated cells. 100% normalized ATP control concentration = 501 pM.

**Figure 8:**
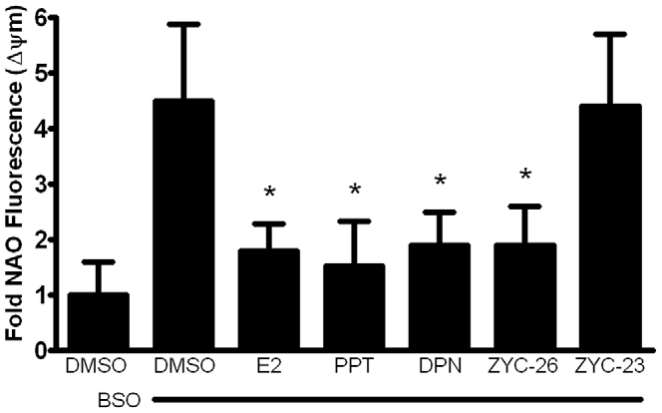
Effects of E2, PPT, DPN, ZYC-26 and ZYC-23 on the collapse of mitochondrial membrane in BSO-treated FRDA fibroblasts. All steroid concentrations were 100 nM, DMSO concentration was 0.1% and BSO concentration was 1 mM. Depicted are mean ± SD for n = 8 per group. * indicated p<0.05 versus BSO alone-treated cells.

It was previously reported that after the application of 1 mM BSO, large-scale cell death occurs at 48 hours (Fig. 3a) [29], [36], with a peak ROS level at 12 hours [29]. Here we have determined a timeline of events beginning with BSO treatment that lead to death in these FRDA fibroblasts. At 12 hours, ROS levels peak at 2–3 fold greater than control, which can be significantly attenuated by co-treatment with estrogens. This is followed by a rise in lipid peroxidation, impairment of aconitase activity, disruption in mitochondrial respiration and reduced ATP content at 24 hours (Figs. 4, 5, 6 and 7) and collapse of the mitochondrial membrane potential (ΔΨm) at 36 hours (Fig. 8), all of which can be partially prevented with estrogen. Finally, at 48 hours there is widespread cell death, which can be significantly reduced by phenolic estrogens (Fig. 3b) [29].

### Effects of steroids on cell viability in BSO-treated FRDA fibroblasts

It has been previously reported that after the application of 1 mM BSO, large scale death occurs at 48 hours (Fig. 3a) [29], [36], with peak ROS levels at 12 hours [29]. To determine the effect of 100 nM E2, PPT, DPN, ZYC-26 and ZYC-23 on BSO-treated fibroblasts, we measured cell viability via a LDH assay (Fig. 3b). BSO decreased cell viability from 96±1% in DMSO-control cells to 47±7%. All of the phenolic steroids provided significant protection at 100 nM, with E2 at 91±1%, PPT at 94±6%, DPN at 94±4% and ZYC-26 at 89±5%. ZYC-23 had no significant effect at 100 nM with an average cell viability of 49±5%, again indicating that the phenolic ring is the crucial part of the estrogen molecule in providing protection in this cell type (Fig. 1).

### Effects of estrogens on lipid peroxidation measurements

To determine the extent of lipid peroxidation produced by BSO-related oxidative stress, 8-isoprostane was measured at 24 hours, in DMSO control and BSO-treated cells in the presence and absence of E2, PPT, DPN, ZYC-26 and ZYC-23 (Fig. 4). BSO increased the level of lipid peroxidation by more than 1.5-fold over DMSO control cells, as measured by intracellular 8-isoprostane concentration at 24 hours. This increase in lipid peroxidation was prevented by each of the phenolic estrogens, but not ZYC-23, indicating that the antioxidant properties of the phenolic rings of these compounds are able to prevent not only ROS accumulation, but also the resulting oxidative damage to intracellular molecules in these cells.

### Effects of estrogens on aconitase activity

Aconitase, an enzyme in the Kreb's cycle responsible for conversion of citrate to isocitrate, is an iron-sulfur containing protein extremely sensitive to reactive oxygen species and has been shown to be significantly inhibited in human FRDA cardiomyocytes, although not in other tissues [6]. Here, we show that in human FRDA fibroblasts there was more than a 50% decrease in aconitase activity in cells treated with 1 mM BSO, and this was partially prevented with co-treatment of all tested phenolic estrogens, but not ZYC-23 (Fig. 5). There was a trend toward compounds with increased numbers of phenolic rings having an increased protective effect in terms of aconitase activity levels. This suggests that estrogens are preventing the loss of aconitase activity by preventing ROS damage to this enzyme.

### Effects of estrogens on mitochondrial respiration

To determine if BSO decreases the level of aerobic ATP production, we assessed the oxygen consumption rate (OCR) with a Seahorse XF-24 metabolic flux analysis (Fig. 6a). BSO-induced oxidative stress significantly decreased both the basal respiratory rate (Fig. 6b) and the maximal respiratory rate (Fig. 6c) suggesting that at 24 hours of BSO treatment permanent damage had already been inflicted on the mitochondria. Interestingly, in the BSO-treated cells, the maximal respiratory rate (45.4±1.0 pMoles/min) was significantly lower than the basal respiratory rate in the DMSO vehicle control cells (50.1±2.0 pMoles/min) (Fig. 6a). Both E2 and ZYC-26 were able to partially offset the decreased respiratory capacity in both basal and maximal states (Fig. 6a). These two estrogens are statistically equivalent in terms of basal respiratory rate rescue, however ZYC-26 appears to be less efficacious at preventing mitochondrial impairment under the maximal state (Figs. 6b and 6c). In all measurements, ZYC-23 provided no significant increase in OCR compared to BSO-alone treated cells. Extracellular acidification rate (ECAR), a measure of anaerobic glycolysis was not statistically altered in any of the four groups (data not shown). Importantly, all of the differences noted in Figures 6a–6c can be attributed solely to mitochondrial dysfunction in living FRDA fibroblasts, as there was no significant cell death present at 24 hours (Fig. 3a). These data indicate that phenol ring containing estrogens are able to partially prevent BSO-induced free radical damage to the mitochondria, independent of any known ER, while non-phenol ring containing estrogens are not.

### Effects of estrogens on cellular ATP concentration

To determine the effect of phenolic estrogens on ATP production, we evaluated each of these estrogen-like compounds with an intracellular ATP concentration assay. Although ATP production and concentration in FRDA fibroblasts is relatively low under baseline conditions compared to other cell types such as primary neurons, BSO significantly reduced the intracellular ATP content by about 35% beyond this level, an effect that was partially prevented by the phenolic estrogens E2, PPT, DPN and ZYC-26, but not the non-phenolic ZYC-23 (Fig. 7).

### Mitochondrial Membrane Potential (ΔΨm) Collapse

To determine the effect of estrogens on mitochondrial membrane potential collapse, an event following ROS-induced mitochondrial damage and preceding cell death, we assessed each of our phenolic and non-phenolic compounds with a FRET assay at 36 hours after BSO treatment, a time point at which there was a peak rise in NAO fluorescence in the BSO treated cells (data not shown). At 36 hours, before significant cell death occurs (Fig. 3a), there was a 4–5 fold increase in NAO fluorescence in BSO treated cells compared with DMSO controls. This effect was prevented by the addition of 100 nM of the phenolic estrogens E2, PPT, DPN and ZYC-26, but not ZYC-23 (Fig. 8). These data indicate that estrogen-like compounds are able to prevent oxidative-stress induced collapse of ΔΨm, an event indicating mitochondrial function disruption that occurs prior to cell death (Fig. 3a), and that this effect is dependent on the presence of a phenol ring in the molecular structure.

### Effects of estrogens on glutathione concentrations

To determine if estrogens were acting to prevent BSO-induced declines in GSH, we assessed GSH concentrations in control cells and those treated with BSO for 24 hours. BSO significantly reduced the levels of GSH below baseline control, and none of the estrogen-like compounds assayed had any effect on GSH concentrations (data not shown). These data show that estrogens are not acting simply to prevent BSO from depleting glutathione or to induce glutathione synthesis *in vitro*.

## Discussion

We have previously reported that 17β-estradiol and other estrogen-like compounds are able to significantly attenuate ROS production and prevent cell death in a human Friedreich's ataxia skin fibroblast model [29]. While the exact mechanism of estrogen neuroprotection is currently unclear, there is mounting evidence that the protective effects of estrogen-related compounds may occur in cells in a nongenomic manner, independent of estrogen receptor activation and subsequent gene expression [24], [46], [47], [48], [49]. The antioxidant properties of estrogens [50] are due to the presence of a phenol at position 3 on the A-ring of estrogens [49], [51]. This phenol ring is responsible for attenuating ROS created by the Fenton reaction *in vitro*, produced in this study using BSO, by a cyclic phenol-quinol mechanism [51].

**Figure 9:**
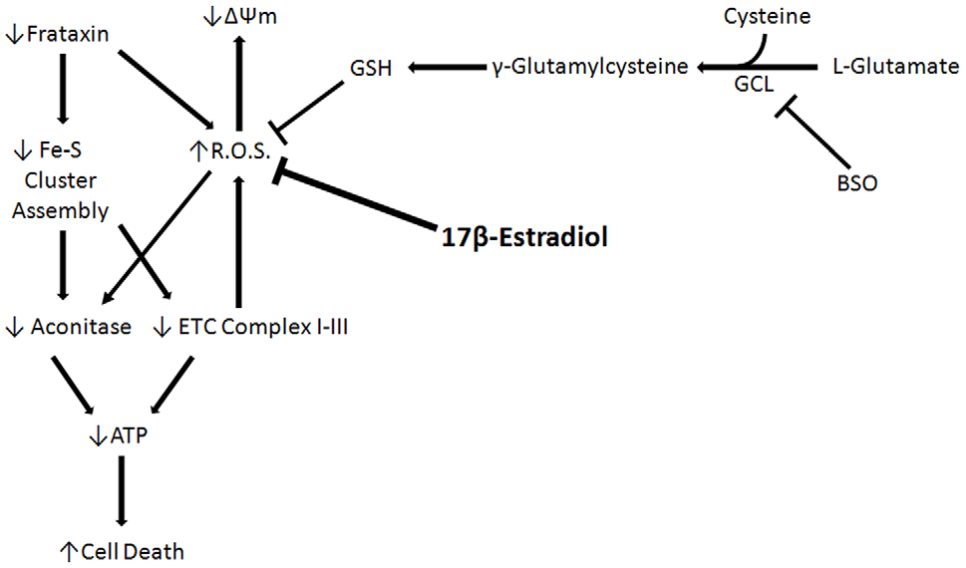
Proposed mechanism of 17β-Estradiol in BSO-treated FRDA fibroblasts.

In the present study, human FDRA fibroblasts were obtained from skin-punch biopsies of a 30-yr-old FRDA patient from Coriell Cell Repositories, a widely accepted cell model for studying FRDA [36], [37], [38], [52]. These cells were homozygous for the FRDA trinucleotide repeat with 541 repeats present on the first allele and 420 present on the second. BSO was used in this model to inhibit *de novo* glutathione synthesis, depleting an important component of these cells' intrinsic defenses against ROS and allowing for the accumulation of ROS produced by natural cell processes, resulting in cell death [29]. The mechanism by which BSO inhibits production of GSH and results in cell death is depicted in Figure 9. Because they are lacking in Frataxin, FRDA fibroblasts are extremely sensitive to BSO-induced oxidative stress compared with normal fibroblasts [36], and thus are used as an *in vitro* model of the long-term consequences of absent Frataxin. Frataxin has been shown to be influential in the production of Fe-S cluster containing proteins [19], [53], [54] and to prevent intracellular ROS rise caused by the toxic effects of excess intracellular iron [18]. In this model, the rise in ROS resulting from the lack of both Frataxin and GSH impairs Fe-S cluster proteins and damages key components of the mitochondria, reducing ATP production and resulting in cell death (Fig. 9).

Here we show that phenolic ring containing estrogens are able to prevent ROS-induced damage of intracellular lipids and proteins, and are able to maintain mitochondrial function despite severe oxidative stress. All of the phenolic compounds tested have been previously shown to prevent an increase in intracellular ROS [29], and they are further able to prevent subsequent damage caused by these ROS. Figures 4 and 5 show that phenolic estrogens prevent oxidative damage to cellular lipids and to the Kreb's cycle protein, aconitase. It has previously been established that E2 is capable of increasing levels of aconitase *in vitro* and *in vivo*, and it is thought that this effect is mediated by a reduction in ROS-related damage to aconitase [55], [56]. Our results indicate that ZYC-26 is able to prevent loss of aconitase activity, while ZYC-23 is not, which argues that E2 increases aconitase activity levels by reducing ROS-mediated damage to this protein in this cell type. Phenolic estrogens also prevent damage to the mitochondria and disruption of their function, as seen by maintenance of oxidative phosphorylation and oxygen consumption rates (Fig. 6), maintenance of near normal ATP levels in cells treated with phenolic estrogens (Fig. 7), and the prevention of ΔΨm collapse (Fig. 8). It has been previously shown that E2 is capable of enhancing overall mitochondrial respiration with the Seahorse assay [57]. In this study we show that mitochondrial function is greatly impaired by BSO, and that the maximal OCR seen in BSO-treated fibroblasts is less than the resting OCR in DMSO control cells (Fig. 6a). The fact that the baseline resting OCR is statistically lower in BSO-treated fibroblasts (Fig. 6b) indicates that there is permanent damage to the mitochondria in these cells at 24 hours after BSO treatment, prior to any cell death (Fig. 3a) [29], [36], [37].This BSO-induced mitochondrial damage can be prevented by either E2 or ZYC-26, but not ZYC-23 in both resting and maximal respiratory states (Figs. 6b and 6c), indicating that the cellular respiratory depression observed in BSO-treated FRDA fibroblasts is due to oxidative damage to the mitochondria and that phenolic estrogens are acting to prevent this oxidative damage in an ER-independent manner. The Seahorse data produced in these experiments corresponds to the decrease in intracellular ATP concentration observed in BSO-treated cells (Fig. 7).

Taken together with results published previously [29], these observations demonstrate that an oxidative insult produces a large increase in reactive oxygen species, leading to consequent lipid, protein and organelle damage, mitochondrial membrane collapse and cell death. Similar to studies in tissue samples from human FRDA patients [6], [19], our results demonstrate that there is significant damage to the mitochondria resulting in the inability of the ATP producing components of the cell to meet energy requirements, leading to large-scale cell death. This can be prevented by the simultaneous application of phenolic estrogens, which effectively reduces the extent of the oxidative insult of individual cells and prevents organelle damage. These data provide a potential mechanism for the protective properties of phenolic estrogens in this system.

In addition, our data support the growing body of evidence that estrogens can act to protect cells and tissues from damage inflicted by neurodegenerative disease by nongenomic means [25], [47], [48], [49]. Since FRDA can be predicted and diagnosed very early [58], [59], [60], there is a window of opportunity to begin treatment in newborns, years before any of the devastating cardiac or neurological symptoms begin to develop, a time period in which non-feminizing estrogen-like compounds may prove to be very efficacious. This study presents the first potential mechanism by which estrogens may be acting to prevent cell death in FRDA and illustrates that non-feminizing estrogens are an attractive class of candidate drugs for the prevention and delay of FRDA symptoms.
